# Antioxidant and Anti-Inflammatory Activities of Flavanones from *Glycyrrhiza glabra* L. (licorice) Leaf Phytocomplexes: Identification of Licoflavanone as a Modulator of NF-kB/MAPK Pathway

**DOI:** 10.3390/antiox8060186

**Published:** 2019-06-20

**Authors:** Luca Frattaruolo, Gabriele Carullo, Matteo Brindisi, Sarah Mazzotta, Luca Bellissimo, Vittoria Rago, Rosita Curcio, Vincenza Dolce, Francesca Aiello, Anna Rita Cappello

**Affiliations:** 1 Department of Pharmacy Health and Nutritional Sciences, Department of Excellence 2018–2022, University of Calabria, Edificio Polifunzionale, 87036 Arcavacata di Rende (CS), Italy; f.luca90@hotmail.it (L.F.); gabriele.carullo@unical.it (G.C.); matteo_brindisi@libero.it (M.B.); mazzottasarah04@gmail.com (S.M.); lucabell92@hotmail.it (L.B.); vittoria.rago@unical.it (V.R.); rosita.curcio@unical.it (R.C.); vincenza.dolce@unical.it (V.D.); 2 Department of Organic and Medicinal Chemistry, University of Seville, C/Prof García González, n. 2, 41012 Seville, Spain

**Keywords:** pinocembrin, licoflavanone, *Glycyrrhiza glabra*, antioxidant activity, anti-inflammatory activity, NF-kB/MAPK pathway

## Abstract

Inflammation represents an adaptive response generated by injuries or harmful stimuli. Natural remedies represent an interesting alternative to traditional therapies, involving several biochemical pathways. Besides, the valorization of agrochemical wastes nowadays seems to be a feasible way to reduce the health spending and improve the accessibility at bioactive natural compounds. In this context, the chemical composition of three *Glycyrrhiza glabra* L. (licorice) leaf extracts, obtained through maceration or ultrasound-assisted method (fresh and dried leaves) was investigated. A guided fractionation obtained three main components: pinocembrin, glabranin and licoflavanone. All the extracts showed similar antioxidant properties, evaluated by 2,2′-diphenyl-1-picrylhydrazyl (DPPH) or 2,2′-azino-bis(3-ethylbenzothiazoline-6-sulfonic acid) Diammonium Salt (ABTS) assay, while, among the isolated compounds, licoflavanone exhibited the best antioxidant activity. The anti-inflammatory activity of the extracts and the purified compounds was investigated in lipopolysaccharide (LPS)-stimulated RAW 264.7 murine macrophages. Extract C and licoflavanone showed a good anti-inflammatory activity without affecting cell viability, as they decreased nitrite levels even when used at 12.5 μg/mL (*p* < 0.005) and 50 μM concentration (*p* < 0.001), respectively. Interestingly, licoflavanone markedly decreased pro-inflammatory cytokines and cyclooxygenase 2/inducible nitric oxide synthase (COX-2/iNOS) expression levels (*p* < 0.001). A modulation of nuclear factor kappa B/mitogen-activated protein kinases (NF-kB/MAPK) pathway underlay such behavior, highlighting the potential of this natural compound as a new scaffold in anti-inflammatory drug research.

## 1. Introduction

Inflammation is a physio-pathological condition characterized by a complex biological response of body tissues to physical, chemical and biological stimuli. This process involves immune cells, blood vessels and molecular mediators in order to remove damage and to start tissue repair but, on the other hand, chronic inflammation can favor the onset of various diseases [1,2]. Over the years, many anti-inflammatory drugs have been developed with the aim to reduce inflammatory mediators’ levels in the body [3]. Most of these molecules act as cyclooxygenase (COX) inhibitors. They belong to a class of drugs that evolved over time to include selective inhibitors of COX-2, the isoform induced in inflammatory conditions, in order to reduce side effects of traditional nonsteroidal anti-inflammatory drugs (NSAID) therapy [4]. In addition, in the last decades, many natural compounds were used as templates for the development of new anti-inflammatory agents [5,6,7]. Plant phenols, particularly flavonoids, feature a wide range of biological activity, mainly including antioxidant and anti-inflammatory activity, useful as alternative medicaments [8,9,10,11]. Many studies highlighted that different phytocomplexes derived from *Glycyrrhiza glabra* L. are endowed with multiple biological activities, including antioxidant and anti-inflammatory activity, which were studied in different experimental models [12,13,14]. Although for a long time the most utilized parts of *G. glabra* were the roots, many studies reported that also its aerial parts, usually treated as an agrochemical waste, contained many bioactive compounds such as dihydrostilbenes [15,16,17], pinocembrin [18,19], licoflavanone, formononetin, isoquercitrin, genistein, prunetin, wighteone, 6-prenylnaringenin and lupiwighteone [19].

Dihydrostilbenes derived from *Glycyrrhiza glabra* leaves are structurally related to flavonoids and exhibit antigenotoxic, antioxidant and anti-inflammatory activity [16,17]. In previous studies, performed on autochthonous *Glycyrrhiza glabra* L., pinocembrin was recovered in leaf extracts, also assayed for their anti-proliferative, antioxidant and antibacterial activities [18,20]. Pinocembrin resulted as the principal component of the total extract, recoverable from methanol fraction after column chromatography. Literature data report that pinocembrin is a flavonoid endowed with antioxidant [21] and anti-inflammatory properties, exerted by downregulating tumor necrosis factor alpha (TNF-α), interleukin 1 beta (IL-1β), and interleukin 6 (IL-6). These activities are also achieved by significantly suppressing the phosphorylation and activation of extracellular signal-regulated kinase (ERK), c-Jun NH2-terminal kinase (JNK) and p38MAPK, as well as by reducing the phosphorylation of IkBα, directly involved in nuclear factor kappa B (NF-kB) regulation, as demonstrated by in vitro and in vivo studies [22]. In *Glycyrrhiza glabra* L. extracts, several bioactive metabolites were also identified [23], including flavonoids able to inhibit the production of nitric oxide (NO), interleukin-6 (IL-6) and prostaglandin E2 (PGE2) in lipopolysaccharide (LPS)-induced macrophages [24]. In this work, an efficient and fast method to recover pinocembrin, an emergent tool in pharmaceutical research [25], was firstly apprehended. For this purpose, ultrasound-assisted extraction (UAE) was applied both to fresh and dried leaves of *Glycyrrhiza glabra*. Furthermore, this procedure allowed us to isolate two prenylated flavanones, known as glabranin and licoflavanone, whose anti-inflammatory activities were also tested. Our outcomes revealed a strong biological potential of licoflavanone, whose molecular mechanisms underlying its anti-inflammatory effects were elucidated for the first time.

## 2. Materials and Methods

### 2.1. Plant Material

Leaves of *Glycyrrhiza glabra* L. were collected in the Botanic Garden, University of Calabria, Rende (Cosenza province, Southern Italy) (39°35′74″ N, 16°22′94″ E) in May 2017. The cultivation generally requires soil 1.4 m deep or more, having a light, loamy and stone-free texture. As a perennial plant, it is grown continuously on the same land, with pieces of stolons planted in March at 0.91 m distance. Two prunings are necessary during the year (in May and October, generally). Fresh leaves recovered in May [18] were used to prepare the extract named B (see later). The remaining leaves were stored at room temperature, in the dark far from light and heat sources, for 5 months before the extraction.

### 2.2. Chemical Reagents and Instrumentation

Ethanol 96%, *n*-hexane, and ethyl acetate (analytical grade) were obtained from Levanchimica srl (Bari, Italy). Silica gel (0.040–0.063 and 0.063–0.200 mm) and thin-layer chromatography (TLC, silica gel plates 60 F254) were acquired from Merck (Darmstadt, Germany). TLC were visualized at 254 nm. Ultrasound-bath Branson model 3800-CPXH (Milan, Italy) was used to perform the extractions. Organic solutions were dried over MgSO_4_ and evaporated on a rotary evaporator (Büchi RII) under reduced pressure. Melting points were obtained using a Gallenkamp melting point apparatus.

### 2.3. Extraction Procedures

**Figure 1:**
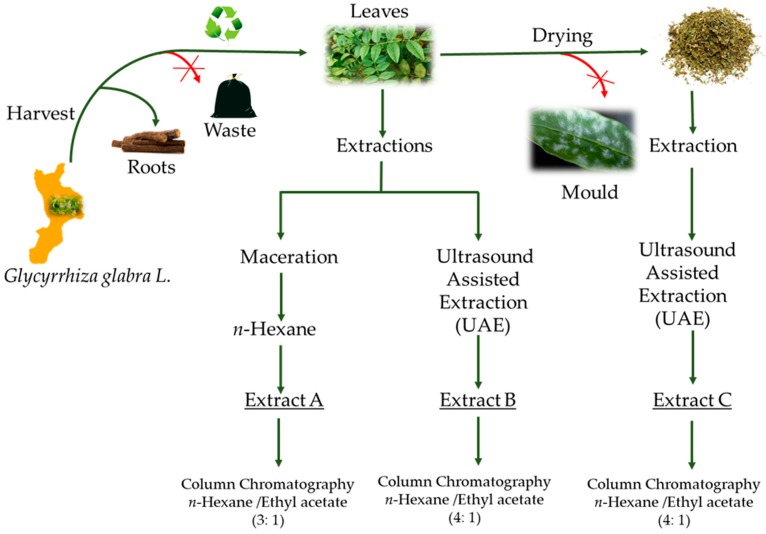
Representative extraction procedures.

The extract A was obtained as previously described [13]. Different procedures were performed to obtain extracts B and C. Fresh or dried leaves (10 g), previously triturated, were extracted with ethanol 96% (250 mL) at 50 °C (10 cycles/s) for 1.5 h, at an ultrasonic frequency of 40 kHz. Leaves were filtered and the solution was evaporated under reduced pressure at 40 °C (Figure 1). The yields of extracts, named B, a greenish gummy resin (fresh leaves) or C, brown gummy resin (dried leaves) were 4.87 and 5.21 g, respectively.

### 2.4. Chemical Characterization and Isolation of the Flavanones

Extracts B and C were analyzed via gas chromatography/mass spectrometry (GC-MS) analysis. After dissolving samples in methanol, GC analyses were achieved by using a Shimadzu GC17AGC equipped with HP 5MS column and a flame ionization detector (FID) controlled by Borwin Software (Shimadzu, Milan, Italy). GC/MS analyses were completed by using a Hewlett-Packard 6890 gas chromatograph connected to a Hewlett-Packard 5973 Mass Selective, using electron impact (EI) (Agilent Technologies, Milan, Italy). Injector and detector temperatures were maintained at 250 °C. The initial oven temperature was set at 50 °C for 3 min. The temperature rate was set on 10 °C min^−1^ up to 250 °C. The extracts (1 g) were subjected to flash column chromatography purification (*n*-hexane/ethyl acetate), in order to isolate the main components. ^1^H-NMR and ^13^C-NMR spectra of the recovered compounds were detailed on a Bruker 300 MHz spectrometer. Chemical shifts (δ) were reported in ppm, using tetramethylsilane (TMS) as the internal reference standard. Coupling constants were reported as a *J* value in Hertz (Hz).

### 2.5. Determination of Total Phenolic Content

The total phenolic content of different licorice extracts was determined using the Folin–Ciocalteu method as previously described [26]. Briefly, 50 µg of each extract (from DMSO solution) was mixed with 333 µL of 1:10 diluted Folin–Ciocalteu reagent, 267 µL of 10% Na_2_CO_3_ was added to the mixture, and a final volume of 1 mL was reached by adding distilled water. Each sample was incubated for 2 h at room temperature and the absorbance at 765 nm was measured by using a UV-Vis spectrophotometer (Ultrospec 2100 Pro, Amersham Biosciences/GE Healthcare). A Gallic acid standard curve was used to estimate total phenolic content of each extract, expressed as mg of gallic acid equivalents per gram of extract.

### 2.6. 2,2′-diphenyl-1-picrylhydrazyl (DPPH) Assay

The 2,2′-diphenyl-1-picrylhydrazyl (DPPH) assay was performed as already reported [27]. Briefly, different concentrations of extracts or isolated compounds were blended with 100 µL of DPPH methanol solution (1 mM), then MeOH was added to make a final volume of 3 mL. Mixtures were vigorously stirred and let stand in the dark for 30 min at room temperature. In each sample absorbance decrease was evaluated at a wavelength of 517 nm, by a UV-Vis spectrophotometer (Ultrospec 2100 Pro, Amersham Biosciences/GE Healthcare).

### 2.7. 2,2′-Azino-bis(3-ethylbenzothiazoline-6-sulfonic acid) Diammonium Salt (ABTS) Assay

The 2,2′-azino-bis(3-ethylbenzothiazoline-6-sulfonic acid) Diammonium Salt (ABTS) assay was carried out as previously described [27,28]. ABTS radical cation (ABTS• +) was attained by reacting ABTS solution (7 mM) with potassium persulfate solution (2.45 mM) and incubating in the dark for 16 h at room temperature before use. The achieved solution was diluted using ethanol in order to reach an absorbance of 0.70 ± 0.05 (monitored at 734 nm). Different concentrations of extracts or isolated compounds were added to 1 mL of ABTS• ^+^ solution and mixtures were incubated under stirring in darkness for 5 min. Absorbance was measured at 734 nm using a UV-Vis spectrophotometer (Ultrospec 2100 Pro, Amersham Biosciences/GE Healthcare).

### 2.8. Cell Cultures

Murine macrophages RAW 264.7 cell line was purchased from the American Culture Collection (ATCC, Manassas, VA, USA) and cultured in Dulbecco’s modified Eagle’s medium (DMEM, Sigma-Aldrich) supplemented with 10% fetal bovine serum (FBS, Sigma-Aldrich), 2 mM L-glutamine (Gibco, Life Technologies) and 1% penicillin/streptomycin (Gibco, Life Technologies) at 37 °C in a humidified atmosphere containing 5% CO_2_. Number of cell passages was kept under 15 from the reference culture (ATCC).

### 2.9. Inhibition of Nitric Oxide (NO) Production in Lipopolysaccharide (LPS)-Stimulated RAW 264.7 Cells

Inhibition of NO production was assessed as previously reported [20]. Briefly, RAW 264.7 cells were seeded at a density of 2 × 10^5^ cells/well in 24-well plates and incubated overnight in complete medium. Media were aspirated and replaced with fresh DMEM, then, cells were simultaneously treated for 24 h with LPS (1 µg/mL) and different concentrations of extracts or isolated compounds. DMSO (Sigma-Aldrich) was used as a vehicle control. After treatment, the presence of nitrite (a stable oxidized product of NO) was measured in cell culture media by using the Griess reagent (Sigma-Aldrich). In brief, 100 µL of cell culture supernatant were incubated with 100 µL of Griess reagent at room temperature for 10 min in a 96-well plate, then, absorbance was measured at 550 nm using a microplate reader (Synergy H1 microplate reader, BioTek).

### 2.10. Cell Viability Assay

Cell viability was assessed quantifying the mitochondrial-dependent reduction of 3-(4,5-dimethyl-2-thiazolyl)-2,5-diphenyl-2H-tetrazolium bromide (MTT) to formazan by living cells, as previously described [29]. Briefly, RAW 264.7 cells were seeded in 24-well plates at the density of 2 × 10^5^ cells/well, and treated with different concentrations of extracts or isolated compounds for 24 h. At the end of the treatment, MTT solution was added to each well (to a final concentration of 0.5 mg/mL) and plates were incubated at 37 °C for 2 h until the formation of formazan crystals. Then, medium was removed, and the formazan precipitate was solubilized in DMSO. In each well, DMSO-solubilized formazan was quantified by reading the absorbance at 570 nm using a microplate reader (Synergy H1 microplate reader, BioTek).

### 2.11. Immuno-Fluorescence Monitoring Nuclear Factor Kappa B (NF-kB) Translocation

RAW 264.7 cells were seeded on coverslip in 6-well plates at a density of 1 × 10^5^ cells/well, and cultured overnight in complete medium. Then, they were treated for 1 h with LPS (1 µg/mL) and extract or licoflavanone, using their IC_50_ values. Next, cells were fixed with ice cold methanol for 20 min at −20 °C, washed three times for 5 min with Tris buffered saline (TBS, Sigma-Aldrich), and incubated for blocking with 5% bovine serum albumin (BSA, Sigma-Aldrich) in TBS for 40 min at 37 °C. Then, cells were incubated for 40 min at 37 °C in anti-NF-kB p65 monoclonal antibody (Santa Cruz, Biotechnology), diluted 1:200. After, they were washed three times for 5 min with TBS to discard the excess of primary antibody, incubated for 40 min at 37 °C in anti-mouse IgG-TRITC (Sigma-Aldrich) diluted 1:300, and subsequently washed three times for 5 min with TBS. Images at 20× magnification were taken on Olympus BX41 microscope with CSV1.14 software, using a CAMXC-30 for image acquisition.

### 2.12. Real-Time RT-PCR Assays

Cells were grown in 10 cm dishes to 70–80% confluence and exposed for 6 h to the vehicle (DMSO) or to LPS (1 µg/mL) alone or in the presence of extract C/licoflavanone at their IC_50_. Total cellular RNA was extracted using TRIZOL reagent (Invitrogen), as suggested by the manufacturer. RNA purity and integrity were assayed spectroscopically, as well as by gel electrophoresis before performing analysis. Complementary DNA (cDNA) was synthesized by reverse transcription as already described [30]. Gene expression analyses of inducible nitric oxide synthase (iNOS), cyclooxygenase 2 (COX-2), tumor necrosis factor alpha (TNFα) interleukin 1 beta (IL 1β) and interleukin 6 (IL 6) were performed using the platform Quant Studio7 Flex Real-Time PCR System (Life Technologies) using SYBR Green Universal PCR Master Mix (BioRad), according to manufacturer’s recommendations. Assays were performed in triplicate and results were normalized using Glyceraldehyde 3-phosphate dehydrogenase (GAPDH) mRNA levels. Relative mRNA levels were calculated using the ∆∆Ct method comparing to control group. All the primers used for amplifications are listed in Table 1.

### 2.13. Immunoblotting Analysis

RAW 264.7 cells were grown to 70–80% confluence and simultaneously treated for 2 h with LPS (1 µg/mL) and extract or licoflavanone at their IC_50_. Then, cells were lysed as previously described [31], using 200 µL of lysis buffer (50 mM Tris–HCl, 150 mM NaCl, 1% NP-40, 0.5% sodium deoxycholate, 2 mM sodium fluoride, 2 mM EDTA, 0.1% SDS) containing a mixture of protease inhibitors (aprotinin, phenylmethylsulfonyl fluoride, and sodium orthovanadate; Sigma-Aldrich) useful for total protein extraction. The same amounts of proteins were resolved on 15% SDS-polyacrylamide gel, transferred to a nitrocellulose membrane and probed with p-p38, p-JNK and p-ERK1/2 specific antibodies (Santa Cruz Biotechnology). Membranes were stripped and incubated with anti-GAPDH antibody in order to confirm equal loading and transfer (Santa Cruz, Biotechnology). The antigen-antibody complex was detected by incubation of the membranes with peroxidase-coupled goat anti-mouse or goat anti-rabbit antibodies and revealed using the ECL System (Amersham Life Science).

### 2.14. Statistical Analysis

Values are expressed as means ± standard deviation of at least three replicate determinations. Statistical analysis was performed by analysis of variance (ANOVA). Normality of each distribution was confirmed by Shapiro–Wilk’s test, while equality of variance was verified by Levene’s test. Tukey’s post-hoc test was used to study differences between groups. A *p* value ≤ 0.05 was considered statistically significant. Non-linear regression analysis was used to generate sigmoidal dose-response curves to calculate IC_50_ values. Data were evaluated using GraphPad Prism 7.

## 3. Results and Discussion

### 3.1. Analysis of the Extracts and Characterization of the Isolated Compounds

**Figure 2:**
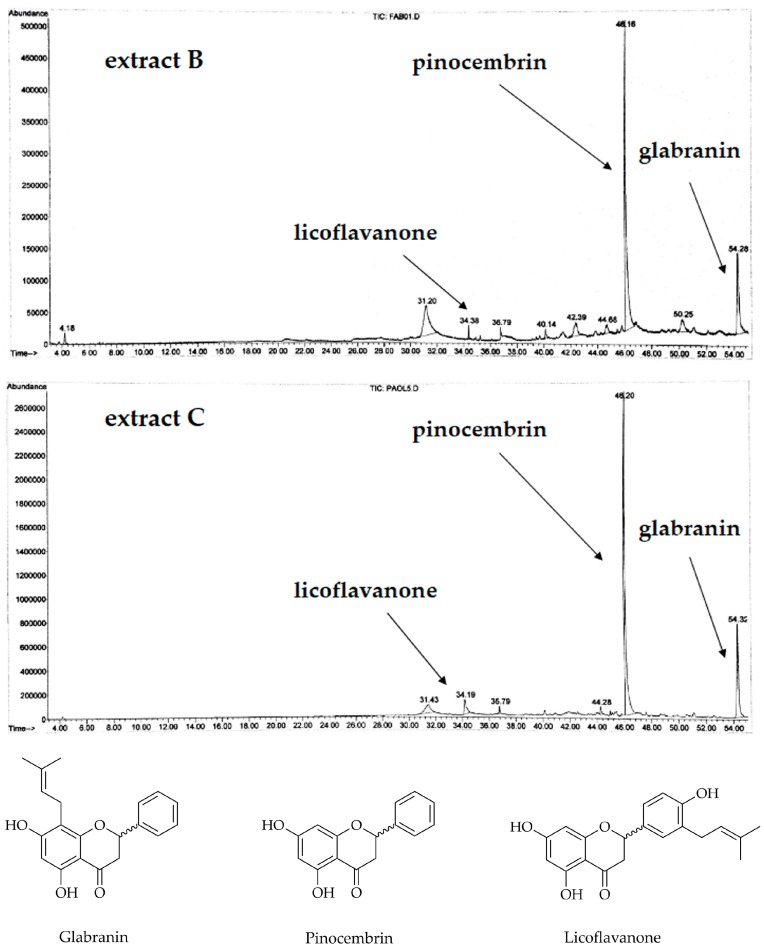
Gas chromatography/mass spectrometry (GC-MS) chromatograms of the phytocomplexes from *Glycyrrhiza glabra* L. leaves and chemical structure of isolated compounds.

The components present in the extracts B and C are summarized in Figure 2. In column chromatography, five visible spots were visualized and isolated. Among them, only spots 2, 3 and 4 were obtained as single compounds, glabranin (M2), pinocembrin (M3) and licoflavanone (M4) (Figure 2), and fully characterized via NMR analysis.

Spot M2. glabranin or (S)-5,7-dihydroxy-8-(3-methylbut-2-en-1-yl)-2-phenylchroman-4-one. Amorphous white solid. clogP: 5.06285. *m/z*: 324 [M^+^]. ^1^H-NMR (CDCl_3_, 300 MHz) δ (ppm) 12.12 (s, 5-OH), 7.59–7.29 (m, 6H), 6.65 (m, 1H), 6.05 (s, 1H), 5.43 (dd, 1H, *J* = 3.1, 12.9 Hz), 5.20 (d, 1H, *J* = 7.0 Hz), 3.33 (d, 1H, *J* = 7.0 Hz), 3.05 (dd, 1H, *J* = 12.9, 17.1 Hz), 2.85 (dd, 1H, *J* = 3.1, 17.1 Hz), 1.80 (s, 6H). ^13^C-NMR (CDCl_3_, 75 MHz) δ (ppm) 196.2, 163.7, 162.2, 159.6, 138.7, 134.6, 128.7, 128.6, 127.9, 125.9, 125.1, 121.6, 106.3, 103.3, 96.9, 78.9, 43.2, 25.7, 21.8, 17.8. These data are in agreement with those reported [32].

Spot M3. (S)-pinocembrin or (S)-5,7-dihydroxy-2-phenylchroman-4-one). White solid m.p.: 198–199 °C. clogP: 3.11185. *m/z*: 256 [M^+^]. Spectroscopic data are in agreement with those reported [13,20].

Spot M4. licoflavanone or (S)-5,7-dihydroxy-2-(4-hydroxy-3-(3-methylbut-2-en-1-yl)phenyl)chroman-4-one. Pale yellow needles m.p.: 134–135 °C. clogP: 4.39585. *m/z*: 340 [M^+^]. ^1^H-NMR (CDCl_3_, 300 MHz) δ (ppm) 12.0 (bs, 1H), 7.28–7.20 (m, 1H), 7.19–7.05 (m, 1H), 6.89 (d, 1H, *J* = 8.3 Hz), 6.05–5.75 (m, 3H), 5.25 (d, 1H, *J* = 3.0 Hz), 3.35 (d, 2H, *J* = 7.3 Hz), 3.08 (dd, 1H, *J* = 12.9, 17.0 Hz), 2.72 (dd, 1H, *J* = 3.0, 17.0 Hz,) H-3), 1.75 (s, 6H). ^13^C-NMR (CDCl_3_, 75 MHz) δ (ppm) 196.8, 164.9, 163.7 (× 2C), 155.0, 131.5 (× 2C), 128.8, 128.5, 124.4, 123.1, 116.2, 102.8, 95.1, 94.6, 82.8, 43.2, 28.1, 24.6, 18.5. Spectroscopic data are in agreement with those reported [23,33,34].

### 3.2. Antioxidant Profile of Glycyrrhiza glabra L. Leaf Extracts and Their Flavanone-Components

**Figure 3:**
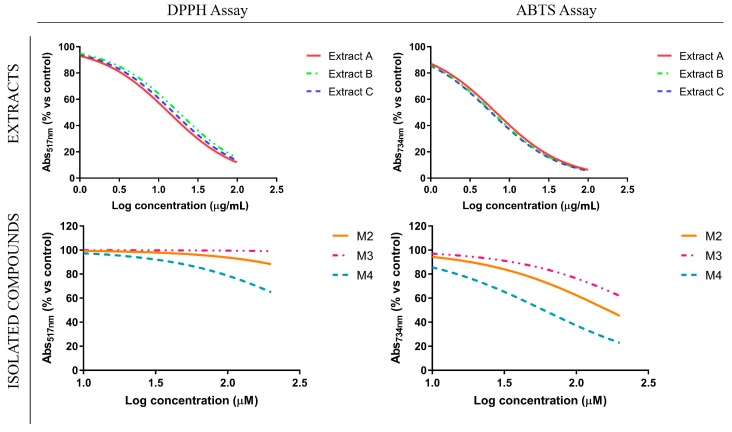
Antioxidant profile of *G. glabra* L. leaf extracts and isolated flavanones.

In order to evaluate and compare the antioxidant power of *Glycyrrhiza glabra* L. leaf extracts A, B, C were subjected to DPPH and ABTS assays. In these experiments, a similar antioxidant profile for the different extracts emerged (Figure 3), with IC_50_ values ranging from 13.49 to 18.05 µg/mL, in DPPH assay, and from 5.88 to 6.76 µg/mL, in ABTS assay (Table 2), a result that reflects the similar total phenolic content estimated for each extract (Table 3). Similar experiments were performed on the three isolated compounds, M2, M3 and M4, to evaluate their contribution to the remarkable antioxidant profile of the extracts. Our results highlighted that the presence of a prenyl group can contribute to the antioxidant profile of flavanones, according to previously reported studies [35]. Indeed, as depicted in Figure 3, the presence of the prenyl group in position 8, characterizing glabranin (M2), improved by itself the poor antioxidant power of its not prenylated analogue pinocembrin (M3). Moreover, licoflavanone (M4) showed the best antioxidant profile, suggesting that either the prenyl group position or the presence of an additional hydroxyl group on the C ring of the flavanone backbone could significantly modify the antioxidant power of these natural products.

### 3.3. Flavanones from Glycyrrhiza glabra L. Leaf Extracts Affect NO Production in LPS-Stimulated RAW 264.7 Cell Line

**Figure 4:**
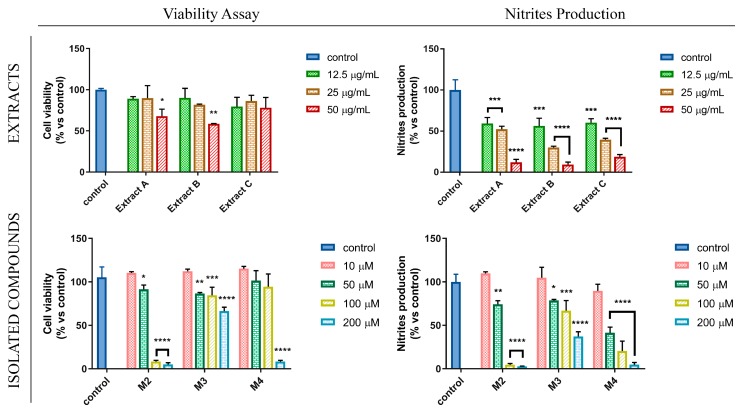
*G. glabra* L. leaf extracts (extracts A, B and C) and the isolated flavanones (M2, M3, M4) modulate nitric oxide (NO) production. Cell viability and nitrites production assessments after treatment of LPS-stimulated RAW 264.7 cell line with different concentration of extracts or isolated compounds for 24 h. MTT assay results are expressed as percentage of cell viability versus control; Griess assay results are expressed as percentage of nitrites production versus control. Values represent mean ± S.D. of three independent experiments, each one performed with triplicate samples. * *p* < 0.05; ** *p* < 0.01; *** *p* < 0.005; **** *p* < 0.001.

A correlation between oxidative stress and inflammation had been already highlighted [36], since several inflammatory stimuli, such as LPS, were able to promote many chronic inflammatory diseases by activating and regulating NO production [37]. In this context, the anti-inflammatory potential of *Glycyrrhiza glabra* L. leaf extracts and their flavanone components were investigated by monitoring their ability to modulate the production of nitric oxide (NO) in LPS-stimulated RAW 264.7 cells, murine macrophages widely used as an in vitro model to study inflammatory pathways. Firstly, we evaluated the effect of increasing concentrations of different *Glycyrrhiza glabra* L. leaf extracts (ranging from 12.5 to 50 µg/mL) as well as their flavanone components (ranging from 10 to 200 µM) on LPS-stimulated RAW 264.7 cell growth, by using MTT assay. These studies displayed that the treatment with extract C did not elicit any significant reduction in cell viability. Conversely, the treatment with extracts A and B reduced cell viability only at the highest tested concentrations. A comparable inhibitory effect on NO production was also shown by using the three extracts at non-toxic concentrations. These findings are in agreement with previous literature data reporting similar anti-inflammatory properties of *Glycyrrhiza* spp. and its bioactive components, tested in our same experimental model [12]. In addition, our results highlighted how the ultrasound-assisted extraction, used in this study, represents a simple and rapid alternative to the maceration process of *Glycyrrhiza glabra* L. leaves, guaranteeing biological activity preservation of the phytocomplex. On the other hand, the effects of the three isolated natural compounds were found to be significantly different from each other, both in terms of cytotoxicity and modulation of NO production. At the tested concentrations, up to 200 µM, pinocembrin showed a poor cytotoxic profile and a non-striking anti-inflammatory activity. On the contrary, the presence of the prenyl group in position 8, characteristic of glabranin, determined a drastic alteration in terms of toxicity/activity ratio, since this compound was found to be highly toxic already when used at 100 µM. Lastly, licoflavanone displayed an intermediate cytotoxicity profile between the two other natural compounds, but it exhibited a significant inhibitory activity on NO production, which was found at much lower concentrations compared to that of the other tested flavanones, reaching an IC_50_ value of 37.68 µM (Figure 4, Table 4). On the basis of these experimental evidences and since no literature data on the pharmacological effects of licoflavanone have been reported so far, we decided to investigate the molecular mechanisms and cellular pathways underlying the promising anti-inflammatory potential of this natural compound.

### 3.4. Licoflavanone Exerts Anti-Inflammatory Effects by Reducing NF-kB Nuclear Translocation

**Figure 5:**
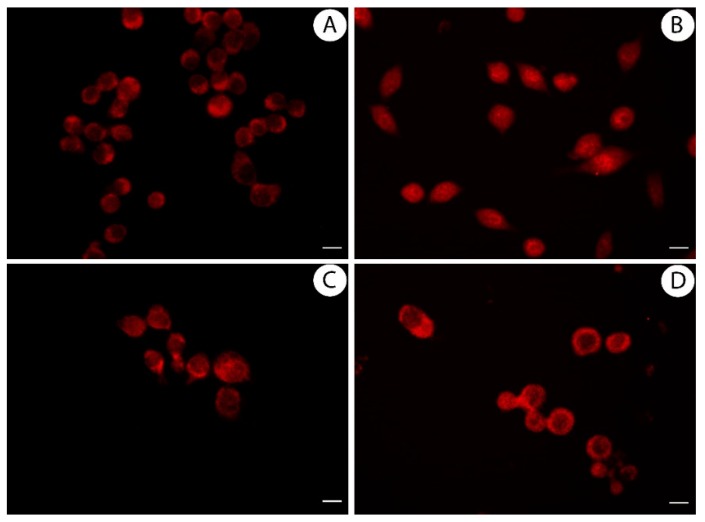
*G. glabra* L. leaf extract and licoflavanone inhibit NF-kB nuclear translocation. Immuno-fluorescent localization of NF-kB in RAW 264.7 cells treated for 1 h with DMSO (**A**), 1 µg/mL LPS + DMSO (**B**), 1 µg/mL LPS + 25 µg/mL Extract C (**C**), 1 µg/mL LPS + licoflavanone at IC_50_ value (**D**). Scale bar: 25 µm.

**Figure 6:**
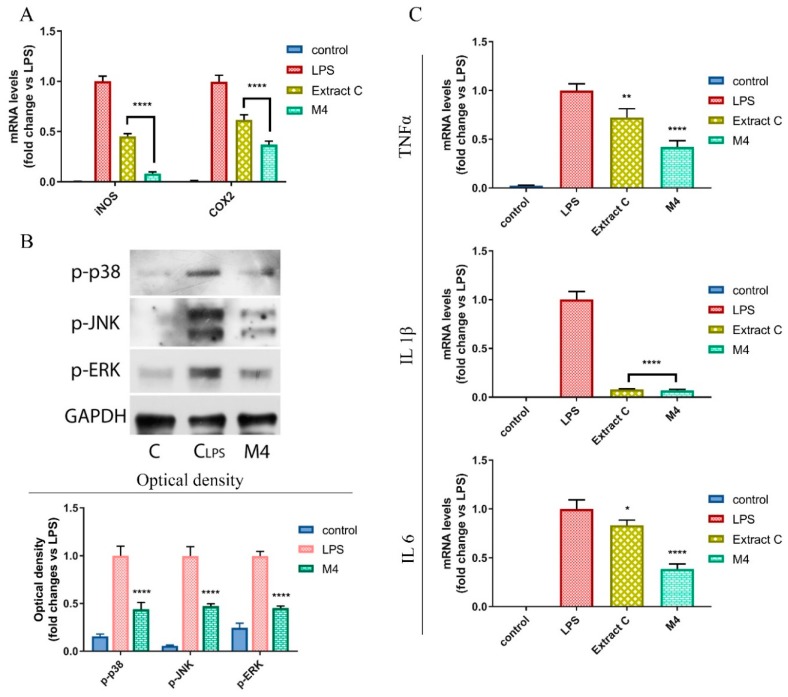
Licoflavanone (M4), as well as the phytocomplex from which it was isolated (extract C), disrupts mitogen-activated protein kinases (MAPK)/NF-kB pathway and modulates pro-inflammatory cytokines in RAW 264.7 cells. (**A**) Effects of extract C and M4 (both used at their IC_50_ values) on inducible nitroxide synthase (iNOS) and cyclooxygenase 2 (COX2) mRNA levels. (**B**) Immunoblotting analysis of phosphorylated MAPKs (p-p38, p-JNK and p-ERK) and relative quantification of expression levels. Glyceraldehyde 3-phosphate dehydrogenase (GAPDH) was used as loading control (**C**) Effects of treatments on pro-inflammatory cytokines (Tumor Necrosis Factor alpha (TNFα), Interleukin-1 beta (IL 1β) and Interleukin-6 (IL 6)) mRNA levels. Treatment conditions for the different experiments are reported in the Methods (Section 2.11 and Section 2.12). Values represent mean ± S.D. of three independent experiments, each one performed with triplicate samples. * *p* < 0.05; ** *p* < 0.01; **** *p* < 0.001.

NO is an inflammatory mediator generated by the catalytic activity of inducible nitroxide synthase (iNOS) enzyme, whose expression is stimulated by inflammatory stimuli such as bacterial LPS through the toll-like receptor 4 (TLR4). The induction of iNOS expression is mediated by NF-kB that is the main transcription factor involved in inflammatory response [38]. Considering the effects exerted by licoflavanone on NO production in LPS-stimulated RAW 264.7 cells, as well as the ability of other flavanones (i.e., pinocembrin) to inhibit inflammatory signal transduction at NF-kB pathway level, we tested the ability of licoflavanone to reduce the activation of this transcription factor. In particular, after the treatment of LPS-stimulated RAW 264.7 cells, the translocation of NF-kB into the nucleus and the expression of two of its target genes, inducible nitroxide synthase (iNOS) and cyclooxygenase 2 (COX2), were monitored. The results obtained in this study highlighted the ability of this natural compound to reduce the translocation of NF-kB into the nucleus (Figure 5) and to significantly decrease the transcription of its target genes (Figure 6A), so excluding that the effects on NO production were due to a direct inhibitory action on iNOS. Such results highlight the significant role of licoflavanone in the inhibition of NF-kB pathway observed for *Glycyrrhiza glabra* L. leaf extract, from which this precious natural product was isolated.

### 3.5. Licoflavanone Disrupts MAPK/NF-kB Pathway and Modulates Pro-Inflammatory Cytokines

The NF-kB pathway is interconnected with that of mitogen-activated protein kinases (MAPKs), which mediate phosphorylation processes that lead to the activation of several transcription factors, such as Activator Protein 1 (AP-1). These factors, together with NF-kB, activate the transcription of different inflammatory genes, including those encoding for the pro-inflammatory cytokines that mediate the amplification and propagation of inflammatory-mediated stress signal. On the basis of NF-kB/MAPKs crosstalk, in this work the ability of licoflavanone to inhibit the cascade of the main MAPKs such as p38, JNK and ERK1/2 was assessed. As shown in Figure 6B, the treatment of LPS-stimulated RAW 264.7 cells with licoflavanone was able to rapidly cause a reduction of p38, JNK and ERK1/2 phosphorylation and activation. Furthermore, the disruption of the NF-kB/MAPKs signal transduction pathway, mediated by licoflavanone, was responsible for a striking reduction in mRNA levels of several pro-inflammatory cytokines, such as Tumor Necrosis Factor alpha (TNFα), Interleukin-1 beta (IL 1β) and Interleukin-6 (IL 6) (Figure 6C). The pharmacological behavior of licoflavanone was found to be in agreement with that of other plant-derived flavanones, confirming that the ability to interfere with the MAPK/NF-kB pathway is a common feature of the different classes of flavonoids. Similar effects, indeed, although in different experimental models, had been described for several flavones, flavonols and flavanones, such as pinocembrin [39], naringenin [40], quercetin [41] and luteolin [42], for which the ability to inhibit MAPKs activation, NF-kB translocation into the nucleus, and the production of pro-inflammatory cytokines, had been reported.

However, some pharmacokinetic limits of flavanoids are their partial absorption at the gastrointestinal level, as well as that they undergo an intensive metabolic process in the liver (methylation, sulfation, and glucuronidation), which favors their elimination, determining their low bioavailability index [43,44,45]. In this regard, since in scientific literature no data are reported on the bioavailability of licoflavanone, it is impossible to know if this natural product could reach, in vivo, the concentrations found to be effective in this in vitro study, therefore, future pharmacokinetic studies will be needed.

## 4. Conclusions

In this report, we provide evidence that three extracts of *Glycyrrhiza glabra* L. leaves, as well as three flavanones (M2, M3 and M4) isolated from them, are endowed with antioxidant properties. In detail, the extracts obtained using different extraction techniques exhibited a similar antioxidant profile, highlighting that UAE is a very rapid method, alternative to the maceration process, able to provide cleaner bioactive phytocomplexes from *Glycyrrhiza glabra* L. leaves. Among the three isolated compounds, licoflavanone (M4) exhibited the best antioxidant activity. On the basis of the already reported link between antioxidant and anti-inflammatory properties [37], we also tested the anti-inflammatory activity of our extracts and isolated flavanones.

Herein, we proved that licoflavanone inhibits LPS-induced expression of TNFα, IL 1β, and IL 6. Furthermore, our outcomes show that licoflavanone is able to decrease iNOS and COX2 expression levels in LPS-stimulated RAW 264.7 cells, interfering with the inflammatory cascade mediated by NO and PGE2. Additionally, in good agreement with literature data reporting that NF-kB pathway is interconnected with that of mitogen-activated protein kinases (MAPKs), our findings evidence that licoflavanone treatment inhibits phosphorylation and activation levels of three important signaling molecules belonging to the MAPK pathway (ERK1/2, JNK, and p38MAPK). Taken together, the present findings prompt us to hypothesize that licoflavanone, gained by ultrasound-assisted extraction from dried leaves of *Glycyrrhiza Glabra* L., possibly exerts its anti-inflammatory effects by interfering with NF-kB/MAPKs crosstalk in LPS-activated macrophages. Nevertheless, further studies will be required to deeply investigate the exact molecular target responsible for licoflavanone action and to confirm its anti-inflammatory effects in in vivo experimental models, which are closer to human ones.

## Figures and Tables

**Table 1:** qPCR primers sequences.

Primer Name	Sequence (5′-3′)
iNOS-Fw	CGAAACGCTTCACTTCCAA
iNOS-Rv	TGAGCCTATATTGCTGTGGCT
COX2-Fw	AACCGCATTGCCTCTGAAT
COX2-Rv	CATGTTCCAGGAGGATGGAG
TNFa-Fw	CAGGCGGTGCCTATGTCTC
TNFa –Rv	CGATCACCCCGAAGTTCAGTAG
IL1b-Fw	GAAATGCCACCTTTTGACAGTG
IL1b-Rv	TGGATGCTCTCATCAGGACAG
IL6-Fw	CTGCAAGAGACTTCCATCCAG
IL6-Rv	AGTGGTATAGACAGGTCTGTTGG
GAPDH-Fw	ACCACAGTCCATGCCATCAC
GAPDH-Rv	TCCACCACCCTGTTGCTGTA

**Table 2:** Antioxidant activity (IC_50_ values) of *G. glabra* L. leaf extracts and isolated flavanones.

	IC_50_ ± SD (µg/mL)			IC_50_ ± SD (µM)		
	Extract A	Extract B	Extract C	M2	M3	M4
**DPPH (IC_50_)**	13.49 ± 1.91	18.05 ± 4.3	15.5 ± 2.5	n.c.	n.c.	n.c.
**ABTS (IC_50_)**	6.76 ± 0.78	6.1 ± 1.04	5.88 ± 0.83	166.3 ± 47.7	n.c.	59.55 ± 21.9

n.c.: not calculable.

**Table 3:** Total phenolic content estimation of *G. glabra* L. leaf extracts.

	Extract A	Extract B	Extract C
**Total phenolic content** (mg Gallic Acid equivalents/g extract)	154.03 ± 10.03	190.37 ± 13.95	165.63 ± 9.21

Extract A: extract from fresh leaves macerated with methanol; Extract B: extract from fresh leaves after ultrasound assisted extraction with ethanol; Extract C: extract from dried leaves after ultrasound assisted extraction with ethanol.

**Table 4:** Nitrites production inhibitory activity (IC_50_ values).

IC_50_ ± SD (µg/mL)			IC_50_ ± SD (µM)		
Extract A	Extract B	Extract C	M2	M3	M4
17.72 ± 3.79	11.73 ± 2.09	16.11 ± 1.5	60.49 ± 26.78	206.5 ± 44.89	37.68 ± 6.67
